# 16. Evaluation of a Monkeypox virus PCR assay and suggestions for future improvement

**DOI:** 10.1093/ofid/ofac492.1867

**Published:** 2022-12-15

**Authors:** Fan Zhang, Courtney Murnane, Ansamma Joseph, Scott Duong, Jamie Lemon, Stefan Juretschko, Vincent Streva

**Affiliations:** Northwell Health Laboratories, Lake Success, New York; Northwell Health Laboratories, Lake Success, New York; Northwell Health Laboratories, Lake Success, New York; Northwell Health Laboratories, Lake Success, New York; Northwell Health Laboratories, Lake Success, New York; Northwell Health laboratories, Little Neck, New York; Northwell Health Laboratories, Lake Success, New York

## Abstract

**Background:**

Monkeypox virus is an Orthopoxvirus in the Poxviridae family currently responsible for an ongoing global epidemic including active transmission in all 50 US states. To date, nearly 12,000 US cases have been confirmed, with the largest case count in New York State, where over 2,500 cases have been confirmed. Of these, the vast majority (greater than 90%) of cases are residents of New York City (NYC). Nearly all described cases have been identified in men, predominantly in men who have sex with men. Until recently, laboratory testing for Monkeypox virus was limited to state and local public health laboratories or the US Centers for Disease Control (CDC). Northwell Health is the largest healthcare provider in the NYC region and is responsible for molecular PCR testing for over 25 hospitals in the area.

**Methods:**

We describe the evaluation of two real-time PCR assays developed by the CDC: one specific for Monkeypox virus and the other a generic Orthopoxvirus assay. We describe performance characteristics in a high-prevalence patient population, identify room for improvement in future assay iterations, and discuss potential scalability of the assay and portability to an ultra high-throughput platform.

**Results:**

We evaluated the CDC Orthopoxvirus and Monkeypox virus PCR assays using 50 clinical specimens collected from patients presenting with lesions. We determined the limit of detection for both assays is 250 copies/mL. Additionally the assays are highly specific with no cross-reactivity with other viruses tested, including HSV-1, HSV-2, and VZV. One significant limitation is the high rate of indeterminate results due to the use of an RNase P specimen control. Indeterminate results were not the result of assay inhibition and may be from inadequate specimen collection. However, since RNase P signal was often absent in positive specimens, the clinical impact of the RNase P result is questionable.

**Conclusion:**

The CDC Orthopoxvirus and Monkeypox virus PCR assays were useful tools for clinical laboratories to launch rapid testing capacity for these viruses early in the course of the outbreak. However, limitations of the assays prevent them from being expanded to more high-throughput testing platforms. Moving forward, additional work is needed to optimize these PCR assays for more widespread clinical use.

**Disclosures:**

**All Authors**: No reported disclosures.

